# Huntingtin-Interacting Protein 1-Related Protein Plays a Critical Role in Dendritic Development and Excitatory Synapse Formation in Hippocampal Neurons

**DOI:** 10.3389/fnmol.2017.00186

**Published:** 2017-06-15

**Authors:** Lin Peng, Qian Yang, Xingxing Xu, Yonglan Du, Yu Wu, Xiaofang Shi, Junyu Xu, Lijun Zhu, Jianhong Luo

**Affiliations:** Key Laboratory of Medical Neurobiology (Ministry of Health of China), Department of Neurobiology, Collaborative Innovation Center for Brain Science, Zhejiang University School of MedicineHangzhou, China

**Keywords:** HIP1R, dendritic development, synapse formation, cultured neurons, proline-rich domain

## Abstract

Huntingtin-interacting protein 1-related (HIP1R) protein is considered to be an endocytic adaptor protein like the other two members of the Sla2 family, Sla2p and HIP1. They all contain homology domains responsible for the binding of clathrin, inositol lipids and F-actin. Previous studies have revealed that HIP1R is highly expressed in different regions of the mouse brain and localizes at synaptic structures. However, the function of HIP1R in the nervous system remains unknown. In this study, we investigated HIP1R function in cultured rat hippocampal neurons using an shRNA knockdown approach. We found that, after HIP1R knockdown, the dynamics and density of dendritic filopodia, and dendritic branching and complexity were significantly reduced in developing neurons, as well as the densities of dendritic spines and PSD95 clusters in mature neurons. Moreover, HIP1R deficiency led to significantly reduced expression of the ionotropic glutamate receptor GluA1, GluN2A and GluN2B subunits, but not the GABA_A_ receptor α1 subunit. Similarly, HIP1R knockdown reduced the amplitude and frequency of the miniature excitatory postsynaptic current, but not of the miniature inhibitory postsynaptic current. In addition, the C-terminal proline-rich region of HIP1R responsible for cortactin binding was found to confer a dominant-negative effect on dendritic branching in cultured developing neurons, implying a critical role of cortactin binding in HIP1R function. Taken together, the results of our study suggest that HIP1R plays important roles in dendritic development and excitatory synapse formation and function.

## Introduction

Huntingtin interacting protein 1-related (HIP1R) protein was first identified due to its structural homology to huntingtin interacting protein 1 (HIP1; Seki et al., 1998). HIP1R, HIP1 and the yeast homolog Sla2p are thought to be endocytic adaptor protein family members that contain homology domains responsible for the binding of clathrin, inositol lipids and F-actin. Unlike HIP1, HIP1R protein does not interact with the huntingtin protein directly (Chopra et al., 2000) and there is no obvious evidence for its involvement in Huntington’s disease (HD). HIP1R knockout mice have no gross morphological abnormalities (Hyun et al., 2004). However, detailed examination of brain development or brain functions has not been performed on those knockout mice. Compared to HIP1 homozygous mice, HIP1R/HIP1 double knockout mice exhibit more serious phenotypes. They show accelerated development and penetrance of abnormal traits, including spinal defects and growth arrest seen in Hip1-deficient mice (Hyun et al., 2004), suggesting a regulatory or compensatory role of HIP1R on HIP1 function. Masuda et al. (2013) examined the expression of HIP1R in the mouse brain at both the protein and mRNA levels, and showed that HIP1R displays a spatially and temporally regulated expression pattern. In addition, Okano et al. (2003) showed by electron microscopy that HIP1R protein is enriched in the synaptic plasma membrane fraction and is subcellularly localized at postsynaptic sites in the rat brain. These data suggest a possible role of HIP1R in the central nervous system, but its function and underlying mechanism of action in neurons remain unknown. In this study, we used shRNA to knockdown HIP1R expression in cultured rat hippocampal neurons and found that HIP1R is critical for dendritic branching and excitatory synaptogenesis during neuronal development.

## Materials and Methods

All animal procedures were performed in accordance with the guidelines of the Zhejiang University Animal Experimentation Committee. This study was carried out in accordance with the recommendations of the Zhejiang University Animal Experimentation Committee.

### HIP1R-shRNA Design

HIP1R shRNA, a 19-nucleotide RNA 5′-GCTGC TGGATGAACAGTTT-3′ corresponding to nucleotides 1881–1899 of rat HIP1R (GenBank accession no: NM_021234), was designed and used to construct a GFP-containing lentivirus-mediated RNA interference vector targeted to HIP1R (HIP1R-shRNA lentivirus) and its plasmid version in pGensile-1 (HIP1R-shRNA plasmid). A 19-nucleotide scrambled RNA 5′-GTGAAACCGGGTGCTTATT-3′ was synthesized and used as a negative control vector.

### DNA Constructs

We used mouse HIP1R homologous cDNA vectors for expression and rescue experiments. The mouse sequence homologous to rat HIP1R shRNA is 5′-GCTGCTG GATGAGCAGTTG-3′. Wild-type full-length cDNA of mouse HIP1R (NM_145070.3) was obtained by PCR amplification using a PrimeScript™ One Step RT-PCR Kit (Takara, Japan) and constructed into vectors pEGFP-C3 and Myc-pRK5, using conventional molecular cloning procedures. A series of N-terminally GFP- or Myc-tagged HIP1R mutants was also constructed that included HIP1R_350–1068_, HIP1R_1–350/766–1068_, HIP1R_1–655_, HIP1R_1–350_, HIP1R_350–655_, HIP1R_766–1068_, HIP1R_766–1017_, and HIP1R_1018–1068_. All these expression constructs were verified by sequencing and tested for normal expression by western blotting.

### Antibodies

The following primary antibodies were used in the described experiments: mouse anti-HIP1R (612118, BD Biosciences Pharmingen), rabbit anti-HIP1R (AB9882, Milipore), mouse anti-GluN2B and rabbit anti-GFP (homemade mAb), rabbit anti-GluN2A (ab133265, Abcam), mouse anti-GluA1 (MAB2263, Millipore), rabbit anti-GluA1 (ab131507, Abcam), mouse anti-Myc (A2814, Santa Cruz), rabbit anti-Myc (ab32027, Abcam), mouse anti-MAP2 (M9942, Sigma-Aldrich), mouse anti-GAD67 (MAB5406, Millipore), rabbit anti-γ-aminobutyric acid A receptor (GABA_A_)-α1 (2583376, Millipore) mouse anti-postsynaptic density (PSD95; MAB1596, Millipore), mouse anti-synaptophysin (S5768, Sigma-Aldrich), and mouse anti-β-actin (A5316, Sigma-Aldrich).

The secondary antibodies used for western blotting were goat anti-mouse IgG-HRP (31460, Pierce) and goat anti-rabbit IgG-HRP (31420, Pierce). The secondary antibodies used for immunostaining were from Life Technologies (Grand Island, NY, USA).

### Primary Hippocampal Neuronal Cultures and Transfections

The procedures used were those described by Wang et al. (2012). Briefly, hippocampus tissue was harvested from embryonic day 17 Sprague-Dawley (SD) rats of either sex, and then gently chopped and digested in 0.5% trypsin for 18 min at 37°C. Dissociated cells were plated at a density of 200–700 neurons/mm^2^ in a 35-mm dish or on poly-L-lysine-coated coverslips.

Hippocampal neurons were cultured in Neurobasal Media (Life Technologies) containing 2% B27 supplement (Life Technologies) and 2 mM Glutamax (Life Technologies). For morphological analysis and synaptic related protein detection, neurons were transfected with appropriate plasmids (2 μg per 35-mm dish) using Lipofectamine LTX&Plus Reagent (Invitrogen) according to the protocol provided by the manufacturer, at day *in vitro* 6 (DIV6) and fixed at DIV12 or later. For live imaging of filopodia, neurons were transfected using calcium phosphate (2–4 μg DNA per 35-mm dish) at DIV5 and detected at DIV8.

### Lentivirus- or Plasmid-Mediated RNAi in Primary Cultured Hippocampal Neurons

HIP1R-shRNA/GFP lentivirus mentioned above and scrambled control-shRNA/GFP lentivirus were made by the Shanghai GeneChem Company (Shanghai, China). Cultured hippocampal neurons at DIV6 were infected with lentivirus vectors, or transfected with plasmid vectors. Six days later, the neurons were used for western blotting or electrophysiological or immunofluorescence staining experiments, respectively.

### Postsynaptic Density (PSD) Fractionation of Rat Hippocampal Tissue

PSD fractionation was conducted on adult rat hippocampus using an adapted protocol (Pacchioni et al., 2009; Lu et al., 2015a).

### Biotinylation Assay

A biotinylation assay was performed as reported previously (Cao et al., 2007; Lu et al., 2015a; Zhang et al., 2015). In general, high density cultured hippocampal neurons at DIV13–15 were rinsed twice with buffer A (1× PBS with 0.5 mM MgCl_2_ and 1 mM CaCl_2_). Cells were then incubated at 4°C with 1 mg/ml sulfo-NHS-SS-biotin in buffer A for 30 min and washed twice (5 min each time) with a quenching agent (50 mM glycine in buffer A). Finally, cells were lysed with RIPA buffer (50 mM Tris, pH 7.4, 1 mM EDTA, 2 mM EGTA, 150 mM NaCl, 1% NP40, 0.5% DOC, 0.1% SDS) after washing three times with buffer A. Biotinylated surface proteins were precipitated with immobilized streptavidin beads and analyzed by western blotting with appropriate antibodies.

### Immunocytochemistry

The methods used for immunostaining were as described previously (Zhang et al., 2013) except for some minor modifications. Cultured hippocampal neurons were fixed using 4% paraformaldehyde in PBS containing 4% sucrose for 15 min, and permeabilized in PBS containing 0.3% Triton X-100 at 4°C. After blocking with 5% bovine serum albumin (BSA) in PBS for 1 h at room temperature, the neurons were incubated with a primary antibody in 5% BSA at room temperature for 1 h, and then with a fluorescent secondary antibody at room temperature for 1 h after washing three times in PBS. The stained neurons were mounted using Molecular Probes (Life Technologies) after washing with PBS and observed under a fluorescence microscope. Images were acquired using a confocal microscope LSM-510 (Carl Zeiss) or spinning disk confocal laser-scanning microscope. For each condition, more than 15 neurons from three independent experiments were analyzed using Imaris software.

### Live Cell Imaging

Cultured hippocampal neurons were transfected with HIP1R-shRNA or GFP control plasmid at DIV5 and observed at DIV8 under a confocal microscope DU-897D-CS0 (Yokogawa). The cultured medium was replaced by extracellular solution (ECS). The concentration of carbon dioxide and the temperature were kept appropriate and constant during the entire experimental process. Pictures were taken at 1 min intervals for 20 min. Meta Morph software was used for statistical analysis of filopodia length and density. Protrusions whose widths were smaller than half of their lengths were defined as “filopodia”. Dendrites 40–100 μm away from the soma were selected for analysis. For tracking filopodia movement, tips of filopodia were recognized and labeled by the Imaris sphere function. The final linear trajectory showed the movement pathway of each filopodia tip.

### Western Blotting

Western blotting was carried out as described previously (Zhong et al., 2012; Qiu et al., 2013; Lu et al., 2015b). Briefly, electrophoresis was performed using 10% SDS polyacrylamide gel. The proteins were transferred onto nitrate cellulose membranes. After blocking in TBST containing 5% BSA, the membranes were incubated with different primary antibodies overnight at 4°C. After washing three times in TBST, the membranes were incubated with horseradish peroxidase-conjugated secondary antibodies for 1 h. The ECL (Pierce) system was used for detection. Blots were analyzed using Quantity One software (Bio-Rad).

### Electrophysiology

Cultured hippocampal neurons were prepared for whole-cell recording at DIV12–14. For miniature excitatory post-synaptic current (mEPSC) and miniature inhibitory post-synaptic current (mIPSC) recording experiments, pipette resistances were 4–6 Mω and the holding potential was −70 mV. Recording was performed at room temperature (22–25°C). For mEPSC, the patch electrode solution contained the following (mM): CsMeSO_4_, 115; CsCl, 20; HEPES, 10; EGTA, 0.6; MgCl_2_, 2.5; Na-phosphocreatine, 10; Na-ATP, 4.0; Na-GTP, 0.3; and QX-314, 1.0; (pH 7.3), and osmolarity was 285–295 mOsmol/kg. The ECS was composed of (mM): NaCl, 147; CaCl_2_, 2.0; KCl, 2.0; HEPES, 10; and glucose, 13; (pH 7.2~7.4). For mEPSC recordings, bicuculline (10 μM) was added to ECS to block GABA_A_ receptor-mediated inhibitory synaptic currents. TTX (1 μM) was added to block Na^+^ channel-mediated potential. For mIPSC, the patch electrode solution contained the following (mM): CsCl, 120; EGTA, 10; MgCl_2_, 1; HEPES, 10; NaCl, 5; and NaGTP, 0.3; MgATP, 3; QX-314, 5; (pH 7.3); and osmolarity was between 300 mosmol^−1^ and 310 mosmol^−1^. The ECS had the following composition (mM): NaCl, 147; CaCl_2_, 2.0; KCl, 2.0; HEPES, 10; glucose, 13; and MgCl_2_, 1.0; (pH 7.2~7.4). For mIPSC recordings, dl-2-amino-5-phosphonovalerate (dAPV, 50 μM) and 6-cyano-7-nitroquinoxaline-2,3-dione (CNQX, 10 μM) were added to block N-methyl D-aspartate receptors (NMDARs) and the L-alpha-amino-3-hydroxy-5-methyl-4-isoxazolepropionic acid receptors (AMPARs), respectively.

### Statistics

Comparisons between two groups were tested using an unpaired two sample *t*-test. A nonparametric KS test was used when the data were not normally distributed. All values are shown as mean ± SEM and plots were prepared using Graphpad Prism 6.0 (GraphPad Software, La Jolla, CA, USA).

## Results

### HIP1R is Ubiquitously Expressed in Rat Brain and Exhibits a Wide Subcellular Localization in Neurons

**Figure 1:**
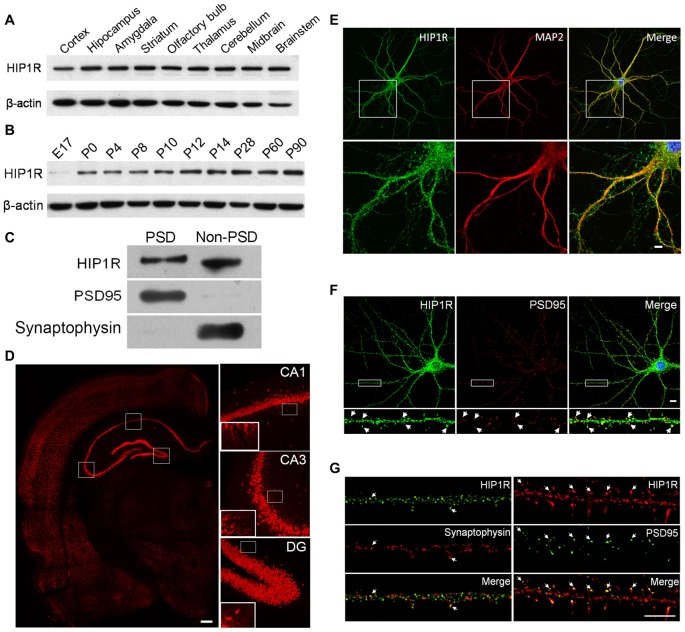
Huntingtin-interacting protein 1-related (HIP1R) expression in rat brain tissues and cultured hippocampal neurons. **(A–C)** Western blotting showed that HIP1R was highly expressed in different brain regions of rats **(A)** expressed with a gradual increase in the hippocampus during postnatal development **(B)** and located partially in the postsynaptic density (PSD) fraction **(C)**. **(D)** Immunohistochemical staining of the hippocampus revealed a strong immunoreactivity of HIP1R in the pyramidal layer in the CA1 and CA3 regions, and the dentate granule cell layer. The insets showed that HIP1R was also distributed on the CA3 and CA1 stratum radiatum. Scale bar = 200 μm. **(E)** Co-immunofluorescence staining of primary cultured hippocampal neurons at days *in vitro* 21 (DIV21) showed that HIP1R exhibited small punctate structures on the soma and along dendrites marked by anti-MAP2 antibody. Scale bar = 10 μm; and **(F,G)** HIP1R puncta were partially colocalized with PSD95, but barely with synaptophysin (arrows). Scale bar = 5 μm. Uncropped images of blots are shown in Supplementary Figure S1.1A–C.

First we examined HIP1R expression in lysates prepared from different regions of the adult rat brain and hippocampus at different developmental stages by immuno-blotting. The HIP1R expression level was considerable and comparable in all brain regions examined (Figure 1A) and gradually increased from the early embryo stage to the adult stage in the hippocampus (Figure 1B). A PSD fractionation assay in hippocampal tissue showed that HIP1R was present in both PSD and non-PSD membrane fractions while the presynaptic marker control synaptophysin was not found in the PSD fraction (Figure 1C).

We then performed immunohistochemical staining on coronal sections of the dorsal hippocampus from 8-week-old rats. The results showed that HIP1R was widely distributed in the hippocampus, with strong signals from the pyramidal layer of CA1 and CA3 regions, and from the cell bodies of the dentate gyrus granule layer (Figure 1D). The insets showed that HIP1R signals were also distributed on the CA3 and CA1 stratum radiatum.

We also examined the subcellular localization of HIP1R in cultured hippocampal neurons at DIV18–21 by its colocalization with MAP2, PSD95 and synaptophysin, using double immune-staining assay. The results showed that HIP1R was expressed as multiple puncta that were widely distributed along dendrites and on the soma (Figure 1E), and partially co-localized with PSD95 clusters (Figure 1F). To characterize its synaptic localization further, ultra-high resolution structured illumination microscopy (SIM) was used to examine colocalization of HIP1R with PDS95 and synaptophysin. The results showed that HIP1R clusters were localized closely behind PSD95 clusters, but barely colocalized with synaptophysin clusters (Figure 1G).

### Knockdown of HIP1R Expression with shRNA Suppresses Dendritic Growth and Spine Formation

**Figure 2:**
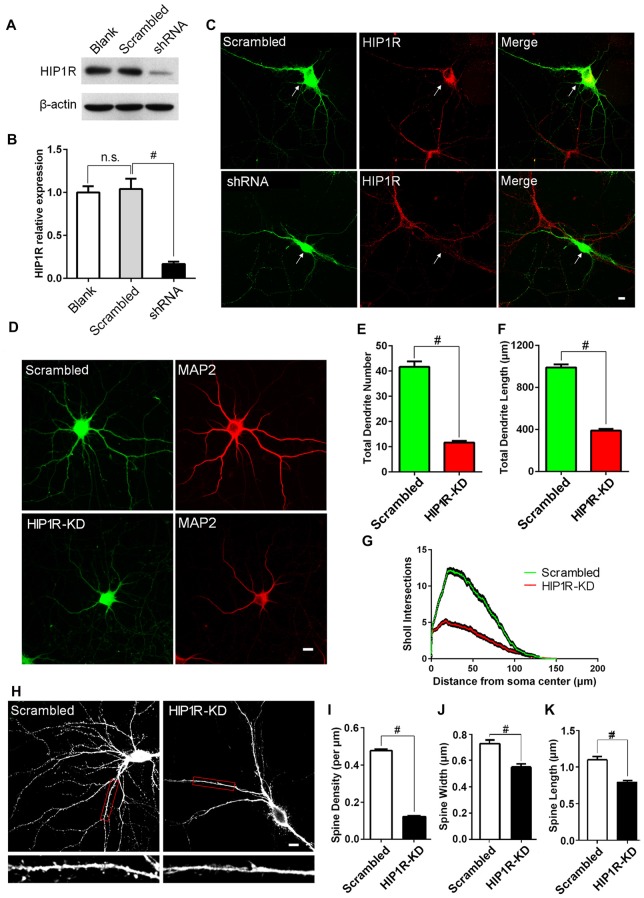
Knockdown of HIP1R expression suppresses dendrite growth and spine formation. **(A)** Cultured hippocampal neurons were infected by HIP1R-shRNA or scrambled control lentivirus and harvested for western blotting at DIV13. A representative western blot shows that the expression level of HIP1R was dramatically reduced in the shRNA group (shRNA) compared to the scrambled control (Scrambled), and viral infection itself had no effect on HIP1R expression (Blank). **(B)** Densitometric analysis of western blotting from 4 independent experiments; Blank, 1.00 ± 0.07; Scrambled, 1.04 ± 0.12; shRNA, 0.17 ± 0.03. **(C)** Cultured hippocampal neurons were transfected at DIV6 with HIP1R-shRNA or scrambled plasmid vectors. Immunofluorescence staining at DIV13 showed a significant decrease in HIPIR immunoreactivity in HIP1R-KD neurons (shRNA) but not in the control (Scrambled). Arrows indicate transfected neurons marked by GFP expression. Scale bar = 10 μm. **(D–G)** Images of HIP1R-shRNA transfected neurons at DIV13 immunostained with anti-MAP2 and used for counting dendrites. Scale bar = 10 μm **(D)**. Unpaired two-tailed *t*-test analysis showed a significant decrease in the total dendritic branch number and total dendritic length in the HIP1R-KD group compared to the scrambled control; 11.69 ± 0.67, *n* = 45 vs. 41.64 ± 2.15, *n* = 45 **(E)** and 388.98 ± 16.63 μm, *n* = 45 vs. 989.91 ± 28.38 μm, *n* = 45 **(F),** respectively. Sholl analysis revealed dendritic complexity dramatically decreased in the HIP1R-KD group compared to the scrambled control (*n* > 40, ^#^*P* < 0.0001) **(G)**. **(H–K)** Images of HIP1R-shRNA transfected neurons at DIV21 immunostained with anti-GFP and used for counting dendritic spines. Scale bar = 10 μm **(H)** The spine density, spine width and length were also significantly reduced in the HIP1R-KD group compared to the control with spine number per μm: 0.12 ± 0.01, *n* = 48 vs. 0.48 ± 0.01, *n* = 48 **(I)**; spine width: 0.56 ± 0.02 μm, *n* = 62 vs. 0.73 ± 0.03 μm, *n* = 62 **(J)**; and spine length: 0.79 ± 0.03 μm, *n* = 62 vs. 1.09 ± 0.04 μm, *n* = 62 **(K)** respectively. All data are presented as mean ± SEM, ^#^*P* < 0.0001. Uncropped images of blots are shown in Supplementary Figure S1.2A.

As HIP1R was found to be widely distributed in the somas and dendrites and its expression level was developmentally regulated, we hypothesized that HIP1R may play a role in dendritic or synaptic development. We tested our hypothesis using the shRNA knockdown assay in cultured hippocampal neurons. To determine the efficiency of HIP1R-shRNA, cultured hippocampal neurons were infected by HIP1R-shRNA or scrambled control lentivirus. Seven days after infection, the neurons were harvested for immune-blotting analysis. The results revealed that HIP1R expression in the shRNA lentivirus group was reduced to 15%–20% of the scrambled control level (Figures 2A,B). Immunofluorescence staining also showed that HIP1R expression was dramatically reduced in HIP1R-shRNA plasmid transfected neurons compared with those of the scrambled control (Figure 2C). These results indicate that HIP1R-shRNA can effectively knockdown the expression of HIP1R in primary cultured neurons. Meanwhile, Supplementary Tables S1 and S2 demonstrated there is minimum chance for the shRNA to be off-target.

To investigate the role of HIP1R protein in dendritic development, cultured hippocampal neurons were transfected with HIP1R-shRNA plasmid at DIV6 and subjected to immunofluorescence staining at DIV12. MAP2 staining was used for better visualization of the dendritic arbor (Figure 2D). The total dendritic branch number and the dendrite length were significantly reduced in HIP1R knockdown (HIP1R-KD) neurons (Figures 2E,F). Sholl analysis also revealed that dendritic complexity was markedly reduced in HIP1R-KD group compared to the scrambled control (Figure 2G). The dendritic spine density and the average spine width and length analyzed at DIV19–21 were also significantly reduced in HIP1R-KD neurons (Figures 2H–K). These results suggest that HIP1R plays a critical role in dendritic growth and spine formation during neuronal development.

### Over-Expression of HIP1R Promotes Dendritic Growth and Spine Formation

**Figure 3:**
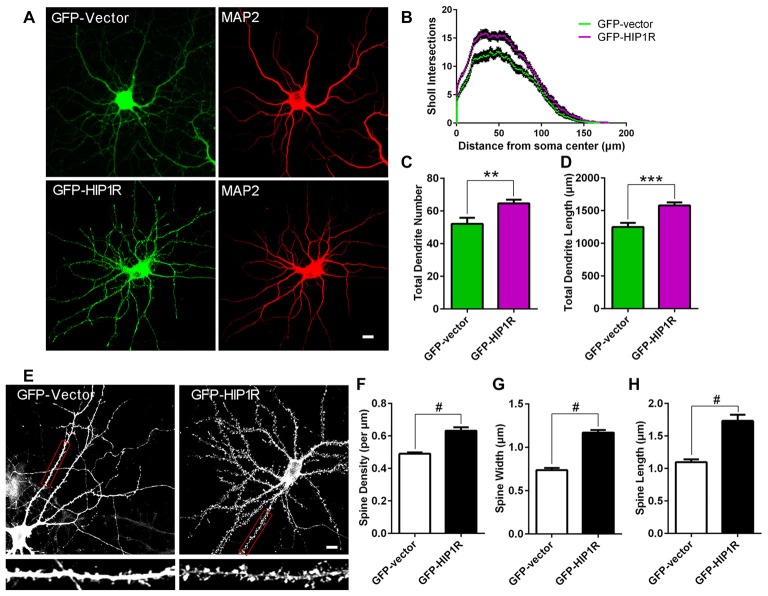
Over-expression of HIP1R enhances dendritic growth and spine formation. **(A–D)** Cultured hippocampal neurons were transfected at DIV6 with GFP-HIP1R or GFP-expressing vectors. Images of the transfected neurons at DIV12 immunostained with anti-MAP2 were used for counting dendrites. Scale bar = 10 μm **(A)** Sholl analysis indicated an increased complexity of dendrites in HIP1R over-expressed neurons (GFP-HIP1R) compared to the control (GFP-Vector) **(B)**. Quantitative data showed that both total dendrite number and total dendritic length were significantly increased in HIP1R over-expressed neurons compared to the control; 64.70 ± 2.326, *n* = 43 vs. 52.17 ± 3.66, *n* = 42 **(C)** and 1518.42 ± 45.80 μm, *n* = 43 vs. 1249.72 ± 65.02 μm, *n* = 42 **(D)**, respectively. **(E–H)** Images of the transfected neurons at DIV21 visualized by anti-GFP immunostaining were used for analyzing dendritic spines by MetaMorph software. Scale bar = 10 μm **(E)**. Quantitative data revealed that spine density, width and length were all increased in HIP1R over-expressed neurons (GFP-HIP1R) compared to the control (GFP-Vector), with spine number per μm: 0.63 ± 0.02 *n* = 48 vs. 0.49 ± 0.01 *n* = 48 **(F)**, spine width: 1.17 ± 0.03 μm, *n* = 61 vs. 0.74 ± 0.03 μm, *n* = 61 **(G)**, and spine length: 1.73 ± 0.09 μm, *n* = 61 vs. 1.10 ± 0.04 μm, *n* = 61 **(H)**, respectively. All data are presented as mean ± SEM, **P < 0.01, ***P < 0.001, ^#^*P* < 0.0001.

To confirm its role in dendritic and synaptic development, we examined neuronal growth after HIP1R overexpression. We transfected GFP-HIP1R expressing plasmids into cultured hippocampal neurons at DIV6–7 and stained the transfected neurons at DIV12 with MAP2 antibody (Figure 3A). Quantitative analysis showed that the total dendrite branch number, total dendritic length and dendritic complexity were significantly increased in GFP-HIP1R overexpressed neurons compared to the GFP control group (Figures 3B–D). The spine density and morphology were also examined (Figure 3E). The results showed that the dendritic spine density and the average spine width and length were all significantly increased in GFP-HIP1R transfected neurons (Figures 3F–H). Together with the knockdown assay, our results indicate that HIP1R functions in dendritic growth and spine formation during neuronal development.

### Knockdown of HIP1R Expression Reduces Dynamics and Density of Dendritic Filopodia

**Figure 4:**
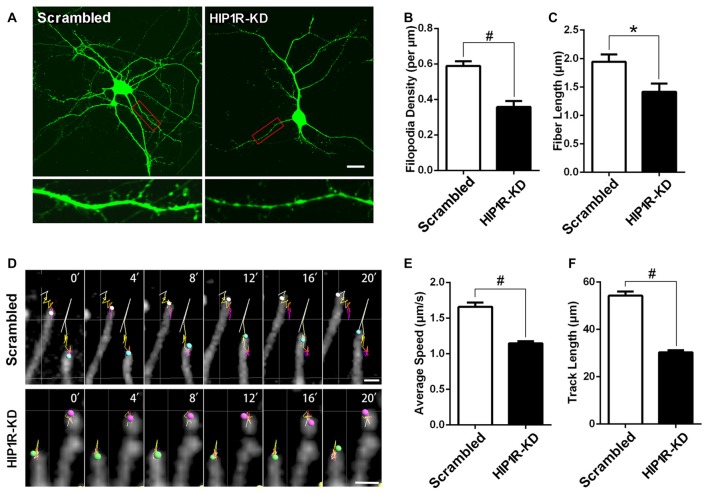
HIP1R knockdown decreases dynamics and density of dendritic filopodia. **(A–C)** Cultured hippocampal neurons were transfected at DIV5 with HIP1R-shRNA and Scrambled vectors. Confocal fluorescence microscopy images were taken at DIV8 and used for analyzing spine morphology by MetaMorph software. Scale bar = 20 μm **(A)**. Quantitative data showed both the density and length of dendritic filopodia were remarkably reduced in HIP1R-KD neurons compared to the scrambled control, with filopodia number per μm: 0.36 ± 0.03, *n* = 30 vs. 0.59 ± 0.03, *n* = 65 **(B)** and filopodia fiber length: 1.42 ± 0.15 μm, *n* = 30 vs. 1.94 ± 0.13 μm, *n* = 65 **(C)**, respectively. **(D–F)** Time-lapse images of living transfected neurons were also taken for analyzing the dynamic activity of filopodia. The linear trajectory shows the movement pathway of each filopodium tip during 20 min and the size of the sphere represents the volume of the filopodium tip each minute. Scale bar = 10 μm **(D)**. The average speed and track length of filopodia tips were obviously reduced in HIP1R-KD neurons compared to those of the control, with average speed: 1.15 ± 0.03 μm/s, *n* = 177 vs. 1.66 ± 0.06 μm/s, *n* = 166 **(E)** and track length: 30.25 ± 0.90 μm, *n* = 177 vs. 54.23 ± 1.76 μm, *n* = 166 **(F)**, respectively. Imaris software was used to track the tips of fibers in each min and analyze speed and track length. All data are represented as mean ± SEM, *P < 0.05, ^#^*P* < 0.0001.

We then evaluated whether HIP1R functions in dendritic filopodia formation and mobility in developing hippocampal neurons. The results showed both the density and length of dendritic filopodia were markedly reduced in HIP1R-KD neurons (Figures 4A–C). Live imaging was carried out to analyze filopodia dynamics by tracking the pathway of each filopodium tip during a 20 min time span. The results revealed that the average speed and track length of filopodia tips were obviously reduced in HIP1R-KD neurons (Figures 4D–F). These data reveal that HIP1R is required for the formation, elongation and mobility of dendritic filopodia in developing neurons.

### Knockdown of HIP1R Decreases Glutamate AMPAR and NMDAR, but not GABA_A_ α1, Subunit Expression

**Figure 5:**
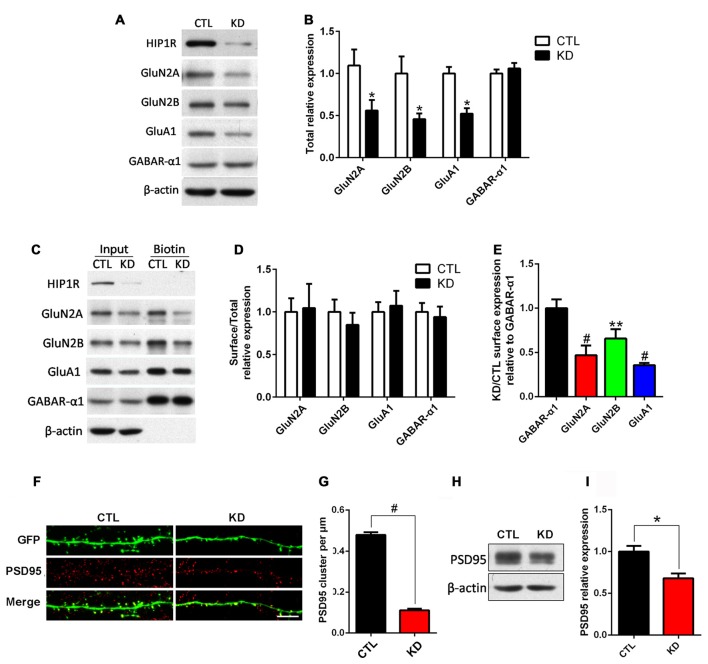
HIP1R knockdown reduces expression of NMDARs, AMPARs and PSD95, but not GABA_A_. Cultured hippocampal neurons were infected with HIP1R-shRNA or Scrambled lentivirus at DIV6 and analyzed by western blotting at DIV13. **(A)** A representative western blot for total GluN2A, GluN2B, GluA1 and GABA_A_-α1 expression. **(B)** Quantification of blots from repeated independent experiments showed that the total expression levels of GluN2A, GluN2B and GluA1 were significantly reduced in the HIP1R-KD group compared to the control with 0.52 ± 0.11, *n* = 7 vs. 1.02 ± 0.18, *n* = 7 for GluN2A; 0.46 ± 0.07, *n* = 6 vs. CTL, 1.00 ± 0.20, *n* = 6 for GluN2B; and 0.52 ± 0.06, *n* = 6 vs. 1.00 ± 0.08, *n* = 6 for GluA1, respectively. **(C)** A representative western blot for surface GluN2A, GluN2B, GluA1 and GABA_A_-α1 expression. **(D)** The ratio of surface to total expression levels of GluN2A, GluN2B, GluA1 and GABA_A_-α1 did not differ between the HIP1R-KD group and the control. **(E)** Surface expression levels of GluN2A, GluN2B and GluA1 were significantly reduced in the HIP1R-KD group compared to the control, when normalized for GABA_A_-α1. There was no significant change in the total and surface expression levels of GABA_A_-α1 in the HIP1R-KD group. The ratio of HIP1R-KD vs. the control: 0.47 ± 0.05, *n* = 4 for GluN2A, 0.66 ± 0.06, *n* = 3 for GluN2B; 0.36 ± 0.01, *n* = 5 for GluA1 vs. 1.00 ± 0.05, *n* = 5 for GABA_A_-α1, respectively. **(F)** Representative images of dendrites immunostained using anti-PSD95 antibody at DIV16 in cultured hippocampal neurons transfected at DIV6 with HIP1R-shRNA and Scrambled vectors, respectively. **(G)** Quantification showed a significant decrease in the cluster density of PSD95 in the HIP1R-KD group compared to the control with 0.12 ± 0.01, *n* = 55 vs. 0.48 ± 0.02, *n* = 55, respectively. **(H)** Representative western blots for PSD95 of lysates from cultured hippocampal neurons at DIV13 6 days after infection with HIP1R-shRNA or Scrambled lentivirus. **(I)** Quantification of western blots from three independent experiments showed decreased expression of PSD95 in the HIP1R-KD group compared to the control with 0.68 ± 0.06, *n* = 3 vs. 1.00 ± 0.07, *n* = 3, respectively. All data are presented as mean ± SEM. *P < 0.05, **P < 0.01, ^#^*P* < 0.0001. Uncropped images of blots are shown in Supplementary Figure S1.5A,C,H.

We assessed whether knockdown of HIP1R influenced the expression of synaptic receptors. Cultured hippocampal neurons were infected with HIP1R-shRNA lentivirus at DIV6–7 and subjected to surface biotinylation and immuno-blotting at DIV12 for receptor detection. The results showed that, in the HIP1R-KD group, the total expression levels of the AMPAR subunit GluA1 and the NMDAR subunits GluN2A and GluN2B were significantly reduced, whereas GABA_A_-α1 expression was unchanged (Figures 5A,B). We also found that the surface expression levels of the GluA1, GluN2A and GluN2B subunits were reduced to a similar extent as the total level (Figure 5E). There were very few changes in surface-to-total expression ratios of GluA1, GluN2A and GluN2B in the KD group compared to those of the control group (Figures 5C,D). Interestingly, both the total and surface levels, and the surface-to-total ratio of GABA_A_-α1 remained unchanged (Figures 5A–E). In addition, excitatory synaptic marker protein PSD95 was reduced, as shown by immunofluorescence staining and immune-blotting (Figures 5F–I). These results suggest that HIP1R is selectively associated with the expression of excitatory synapse components, such as glutamate AMPAR and NMDAR subunits and the postsynaptic scaffold protein PSD95, in neuronal development.

### Reduced HIP1R Expression Selectively Impairs Excitatory Synapse Function

**Figure 6:**
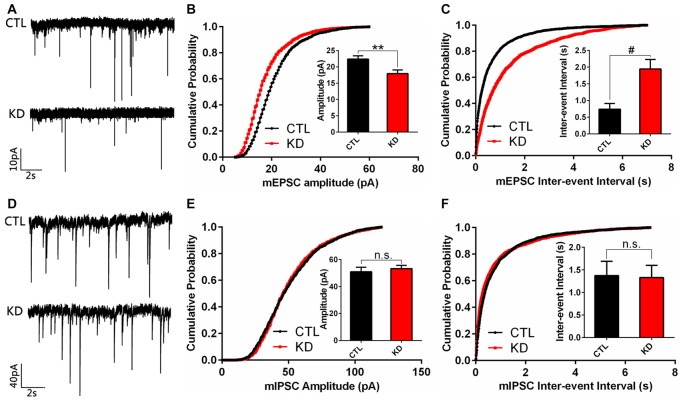
Amplitude and frequency of miniature excitatory post-synaptic current (mEPSC), but not miniature inhibitory post-synaptic current (mIPSC), are reduced in HIP1R-knockeddown neurons. Cultured hippocampal neurons were infected by HIP1R-shRNA or Scrambled lentivirus at DIV6, and whole-cell patch-clamp recording was carried out at DIV13. **(A)** Example traces of mEPSC. **(B)** Graph of cumulative probability and bar graph of amplitude mEPSC (HIP1R-KD, 17.93 ± 1.13 pA, *n* = 19 vs. the control, 22.38 ± 1.03 pA, *n* = 22) and **(C)** Graph of cumulative probability and bar graph of inter-event interval of mEPSC (HIP1R-KD, 1.95 ± 0.28 s, *n* = 19 vs. the control, 0.74 ± 0.17 s, *n* = 22) showed a significant reduction in mEPSC amplitude and frequency in HIP1R-KD neurons compared to the control. **(D)** Example traces of mIPSC. **(E)** Graph of cumulative probability and bar graph of amplitude mIPSC (HIP1R-KD, 53.33 ± 2.32 pA, *n* = 25 vs. the control, 50.98 ± 3.36 pA, *n* = 21), and **(F)** graph of cumulative probability and bar graph of inter-event interval of mIPSC (HIP1R-KD, 1.33 ± 0.27 s, *n* = 25 vs. the control, 1.37 ± 0.31 s, *n* = 21) did not show marked differences in mIPSC amplitude and frequency between the two groups. All data are presented as mean ± SEM, unpaired two-tailed *t*-test. **P < 0.01, ^#^*P* < 0.0001.

As HIP1R selectively regulates the expression of excitatory ionotropic glutamate receptors, we examined if reduced HIP1R expression also preferentially affects synaptic transmission in cultured hippocampal neurons. Whole cell patch-clamp recording was carried out in shRNA-infected neurons at DIV12. The results showed that both the mEPSC amplitude and frequency were dramatically reduced in the HIP1R-KD group (Figures 6A–C). In contrast, neither the amplitude nor frequency of mIPSC differed between the KD and control groups (Figures 6D–F). These results demonstrate that HIP1R is associated mainly with the formation and function of excitatory synapses.

### Mouse HIP1R Re-Expression Rescues Dendritic Growth Defects in HIP1R-Knocked down Neurons

**Figure 7:**
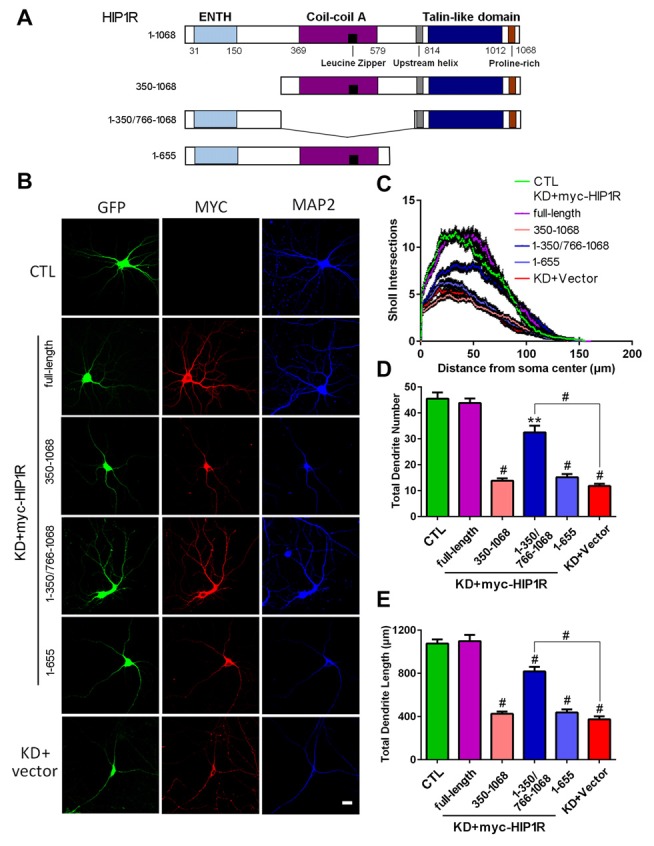
Transfection of mouse HIP1R rescues the dendrite growth defects in HIP1R-knockeddown neurons. **(A)** A schematic illustration showing mouse full-length and several truncated forms of HIP1R cDNA in the myc-tagged expressing vector. **(B)** Cultured hippocampal neurons at DIV6 were co-transfected with HIP1R-shRNA for knocking down endogenous HIP1R expression, and full-length or truncated HIP1R vectors for re-expressing exogenous cognates. Transfected neurons were immunostained with myc and MAP2 antibodies at DIV12. GFP-expressing and positively myc stained neurons were used for morphological analysis. **(C)** ANOVA analysis with repeated measures of Sholl analysis showed that full-length HIP1R re-expression fully rescued the complexity impairment in HIP1R KD neurons. HIP1R_1–350/766–1068_ had a partial rescue effect. **(D,E)** Unpaired two-tailed *t*-test analysis of total dendrite length and number. Total dendrite length: 1076.65 ± 36.67 μm, *n* = 31 for the control group (CTL); 1098.99 ± 56.12 μm, *n* = 32 for KD+myc-HIP1R; 425.94 ± 20.50 μm, *n* = 33 for KD+myc-HIP1R_350–1068_; 817.70 ± 42.27 μm, *n* = 36 for KD+myc -HIP1R_1–350/766–1068_; 437.96 ± 28.07 μm, *n* = 37 for KD+myc-HIP1R_1–655_; and 374.80 ± 26.39 μm, *n* = 31 for KD + Vector. Total dendrite number: 44.45 ± 2.99, *n* = 31 for the control group (CTL); 43.80 ± 1.81, *n* = 32 for KD+myc-HIP1R; 13.85 ± 0.93, *n* = 33 for KD+myc-HIP1R_350–1068_; 32.53 ± 2.56, *n* = 36 for KD+myc-HIP1R_1–350/766–1068_; 15.19 ± 1.22, *n* = 37 for KD+myc-HIP1R_1–655_; and 11.83 ± 0.83, *n* = 31 for KD + Vector. All data are presented as mean ± SEM. **P < 0.01, ^#^*P* < 0.0001.

To investigate the specificity of the KD effect and potential roles of different domains of HIP1R in dendritic growth, we transfected mouse homologous HIP1R vectors in rat HIP1R-shRNA lentivirus induced HIP1R-KD hippocampal neurons in rescue experiments (Figure 7A). We found that mouse full-length HIP1R re-expression fully rescued the dendrite branching defects, including dendrite number, total length and complexity in HIP1R-KD neurons (Figures 7B–E). This provides further evidence for the specificity of the effect of rat HIP1R-shRNA. However, neither N-terminus-truncated HIP1R_350–1068_ nor C-terminus-truncated HIP1R_1–655_ fragments had such rescue effects. Interestingly, re-expression of HIP1R_1–350/766–1068_, an N- and C-terminal fusion mutant without the central coil-coil domain, partially rescued these defects (Figures 7B–E). These results revealed the role of HIP1R in dendrite growth and its functional dependency on an intact structure.

### Expression of HIP1R C-Terminus or its Proline-Rich Region Confers a Dominant Negative Effect on Dendrite Development

**Figure 8:**
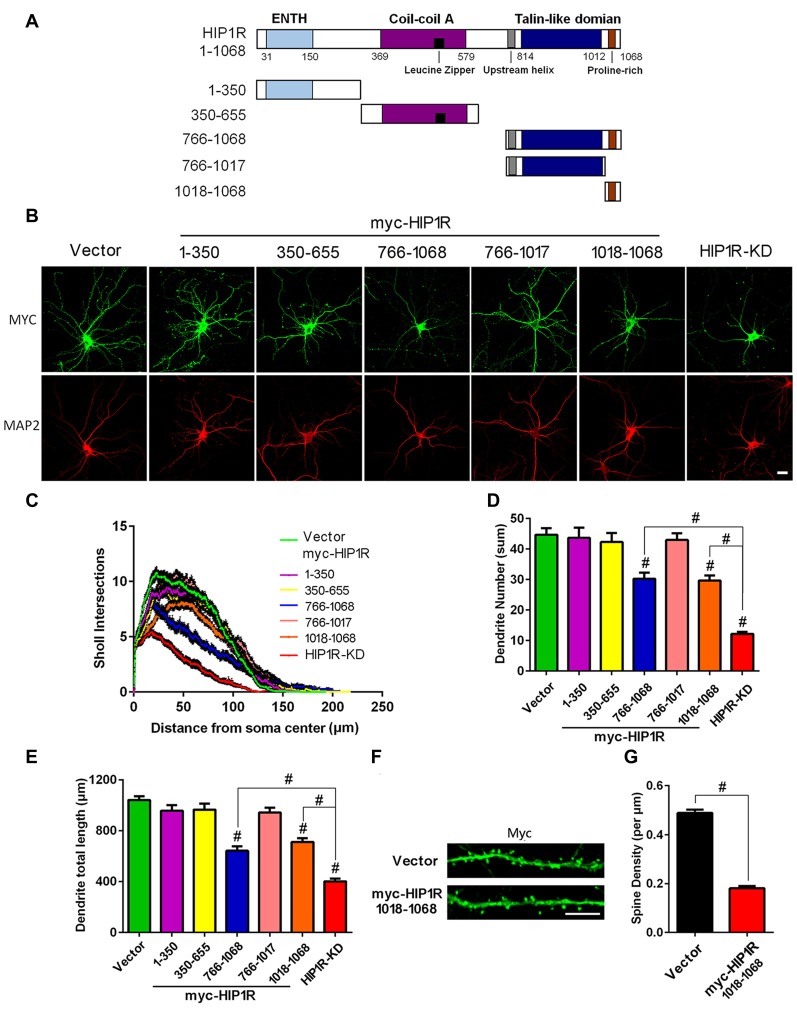
Expression of the HIP1R C-terminus and its proline-rich region confer a dominant negative effect on dendrite development. **(A)** A schematic illustration showing full-length and several truncated forms of HIP1R cDNA in the myc-tagged expressing vector. **(B)** Cultured hippocampal neurons were transfected at DIV6 with each of the myc-tagged HIP1R mutants. An empty vector was taken as a negative control and GFP-shRNA as a positive control. Representative images of transfected neurons are shown immunostained at DIV12 with myc and MAP2 antibodies. Scale bar=20 μm. **(C)** ANOVA with repeated measures for Sholl analysis showed a significant decrease in dendrite complexity in HIP1R_766–1068_ and HIP1R_1018–1068_ transfected neurons. **(D,E)** Quantitative analysis with unpaired two-tailed *t*-tests showed a partial decrease in dendrite number and total length in HIP1R_766–1068_ and HIP1R_1018–1068_ transfected neurons compared to the negative and positive controls. Dendrite number: 44.69 ± 2.18, *n* = 42 for the negative control (CTL); 43.70 ± 3.30, *n* = 46 for myc-HIP1R_1–350_; 42.30 ± 2.95, *n* = 44 for myc-HIP1R_350–655_; 30.21 ± 2.03, *n* = 48 for myc-HIP1R_766–1068_; 42.98 ± 2.23, *n* = 42 for myc-HIP1R_766–1017_; 29.70 ± 1.59, *n* = 40 for myc-HIP1R_1018–1068_; 12.17 ± 0.68, *n* = 42 for the positive control (KD). Dendrite total length: 1041.91 ± 29.58 μm, *n* = 42 for the negative control (CTL); 957.21 ± 42.99 μm, *n* = 46 for myc-HIP1R_1–350_; 965.05 ± 47.24 μm, *n* = 44 for myc-HIP1R_350–655_; 642.64 ± 34.09 μm, *n* = 48 for myc-HIP1R_766–1068_; 942.51 ± 37.93 μm, *n* = 42 for myc-HIP1R_766–1017_; 711.39 ± 30.27 μm, *n* = 40 for myc-HIP1R_1018–1068_; 401.25 ± 21.66 μm, *n* = 42 for the positive control (KD). **(F,G)** Myc-vector or myc-HIP1R1018–1068 plasmid was transfected into DIV6–7 hippocampal neurons. Transfected neurons were stained with anti-myc antibody to analyze the spine density at DIV18–21. Spine density per μm: 0.18 ± 0.01, *n* = 55 for myc-HIP1R1018–1068 vs. 0.49 ± 0.01, *n* = 55 for the vector control. All data are presented as mean ± SEM. ^#^*P* < 0.0001.

To identify the functional domains of HIP1R critical for dendrite development, we generated a series of myc-tagged HIP1R mutant-expressing plasmids (Figure 8A). Each mutant contains one dominant domain of HIP1R. The mutant-transfected neurons were co-immunofluorescence stained with myc and Map2 antibodies at DIV12 to observe negative dominant effects on dendrite development (Figure 8B). The results showed that only the proline-rich region-containing mutants HIP1R_766–1068_ and HIP1R_1018–1068_ partially reduced the dendrite number, total length and complexity, while the other mutants, HIP1R_1–350_, HIP1R_350–655_ and HIP1R_766–1017_, had no such effects (Figures 8C–E). The dendritic spine density was also significantly reduced in the transfected neurons with the proline-rich region expressing vector (HIP1R_1018–1068_) at DIV21 (Figures 8F,G). These results suggest that the proline-rich region is critical for HIP1R function in dendrite growth and synaptic formation.

## Discussion

In this study, using RNAi techniques we demonstrated that an endocytic adaptor protein HIP1R plays a critical role in dendrite growth and excitatory synapse formation and function in primary cultured hippocampal neurons. We found that knockdown of HIP1R expression significantly reduced dendrite number, total length and complexity as well as the spine density of developing hippocampal neurons in culture. Interestingly, such HIP1R knockdown selectively inhibited expression of glutamate AMPARs and NMDARs and impaired excitatory synaptic transmission. We suggest that HIP1R may play an important role in neural development and synaptic function in mammals.

Recently, it was reported that HIP1R is ubiquitously expressed in different tissues of mice, and exhibits a region-specific expression pattern in the developing and mature brain. This spatial and temporal expression pattern indicated a possible role for HIP1R in neural development (Masuda et al., 2013). HIP1R protein was also found to be enriched in the synaptic plasma membrane fraction and localized in the small vesicular structures in postsynaptic spines, suggesting that HIP1R may play a role in synaptic function (Okano et al., 2003). In this study, using western blotting, immunohistochemistry, and immunofluorescence microscopy, we examined HIP1R expression in different rat brain tissues and cultured neurons and provided further evidence for histological and subcellular localization of HIP1R. Importantly, using RNAi techniques to reduce HIP1R expression effectively in primary cultured hippocampal neurons, we provide for the first time evidence for HIP1R functioning in dendrite growth, branching and spine formation. This major conclusion is supported by our results showing that HIP1R over-expression promotes dendrite growth, and that HIP1R re-expression rescues dendritic growth defects in HIP1R-KD cultured neurons. In addition to the reduced dendritic spine density, we found that HIP1R knockdown leads to a significant lowering of PSD95, AMPAR and NMDAR expression, and therefore, a decrease in the amplitude and frequency of mEPSC. Interestingly, neither the inhibitory receptor GABA_A_-α1 subunit expression nor mIPSC were affected by HIP1R knockdown, suggesting a specific role of HIP1R in excitatory synapse formation and function.

HIP1R has been identified as a member of the Hip1/HIP1R/Sla2p endocytic adaptor protein family that is closely associated with clathrin-mediated endocytosis (Seki et al., 1998). They all have several homology domains responsible for binding to clathrin, inositol lipids and F-actin (Engqvist-Goldstein et al., 1999; Wilbur et al., 2008). A previous study revealed that Sla2p, the yeast homolog of Hip1R, mediates membrane–actin coupling during endocytosis in yeast cells (Skruzny et al., 2012). It is known that endosome trafficking and actin organization are critical for the organization, development, and function of morphologically complex neurons. A variety of endocytic adaptor proteins have been identified (Cosker and Segal, 2014). Previous studies suggest that reducing HIP1R levels using siRNA alters endocytosis and causes abnormal cytoskeletal actin organization (Engqvist-Goldstein et al., 2004; Gottfried et al., 2010). Cortactin is another adaptor protein critical for multiple neuronal functions including cell motility, endocytosis, and cytoskeletal remodeling (Cosen-Binker and Kapus, 2006). It is reported that HIP1R and cortactin form a complex contributing to the regulation of actin assembly in endocytic sites in HeLa cells through direct interaction of the HIP1R C-terminus proline-rich region (a.a. 1018–1068) and the SH3 domain of cortactin (Engqvist-Goldstein et al., 2001; Brett et al., 2006; Le Clainche et al., 2007). In neurons deficient in cortactin, the spine density is greatly diminished, whereas over-expression of cortactin causes elongation of spines (Hering and Sheng, 2003). Interestingly, we found a similar phenomenon by knocking down or over-expressing HIP1R in hippocampal neurons. Moreover, knockdown of cortactin protein results in depletion of dendrites (Hering and Sheng, 2003). In our study, we found that the HIP1R C-terminus or its proline-rich region confers a dominant negative effect on dendrite development, suggesting that HIP1R-cortactin interaction-associated endocytic processes may be involved in dendrite development. It would be interesting to explore further the mechanism of HIP1R function in neuronal development and excitatory synaptogenesis in the future.

## Author Contributions

LP, LZ and JL designed the study. LP, QY, YW and XS performed biochemical experiments. XX and QY constructed vectors. YD performed electrophysiology recording. LP analyzed the majority of the data. LP, QY, JX, LZ and JL wrote the article.

## Conflict of Interest Statement

The authors declare that the research was conducted in the absence of any commercial or financial relationships that could be construed as a potential conflict of interest.

## Supplementary Material

FIGURE S1 All raw blots show both molecular weights and the blot insets for each main figure.
