# Supplementary material for: Huntingtin-Interacting Protein 1-Related Protein Plays a Critical Role in Dendritic Development and Excitatory Synapse Formation in Hippocampal Neurons
Source: Front Mol Neurosci. 2017 Jun 15;10:186. doi: 10.3389/fnmol.2017.00186 (PMC5471304; doi:10.3389/fnmol.2017.00186)
Supplement: Supplementary file 1 [file Table_1.pdf]

Table 1

Nucleotide blast results of Hip1R-shRNA

gene name	Gene ID	Query cover	E value	Identities
Hip1r	ENSRNOG00000001091	100%	0.003	100%
Rai14	ENSRNOG00000028872	89%	0.83	100%
Spidr	ENSRNOG00000037851	100%	0.83	95%
Mex3d	ENSRNOG00000030830	94%	3.3	94%
Tbcld2	ENSRNOG00000023348	89%	13	94%
Xirp2	ENSRNOG00000034258	100%	13	100%
LOC102550632, ncRNA[a]	N.A.	100%	13	100%

[a]Gene ID of LOC102550632 was not found in NCBI.

Table 2

Transciptome Sequencing after HIP1R knockdown

gene name	CTL-Expression	KD-Expression	CTL-RPKM	KD-RPKM	log2 Ratio	Regulation(KD/CTL)	P-value(KD/CTL)	FDR
Hip1r	2402	1930	19.57415511	16.36803267	-0.258069107	Down	4.47E-09	2.48E-07
Rai14	164	160	1.25301543	1.272218497	0.021942287	Up	0.890164	0.958719284
Spidr	32	33	0.400193771	0.429500267	0.101960316	Up	0.775164	0.887435864
Mex3d	1295	1237	19.40696029	19.29241522	-0.008540401	Down	0.882076	0.953218283
Tbcld2	6	3	0.050036017	0.026036457	-0.942433803	Down	0.377456	0.625567851
Xirp2[a]	N.A.	N.A.	N.A.	N.A.	N.A.	N.A.	N.A.	N.A.
LOC102550632, ncRNA[a]	N.A.	N.A.	N.A.	N.A.	N.A.	N.A.	N.A.	N.A.

[a]The mRNA products of these two genes were too low to be detected.
RPKM: Reads Per Kilobases per Million reads
FDR: False Discovery Rate
