# Supplementary figures and images for: Huntingtin-Interacting Protein 1-Related Protein Plays a Critical Role in Dendritic Development and Excitatory Synapse Formation in Hippocampal Neurons
Source: Front Mol Neurosci. 2017 Jun 15;10:186. doi: 10.3389/fnmol.2017.00186 (PMC5471304; doi:10.3389/fnmol.2017.00186)

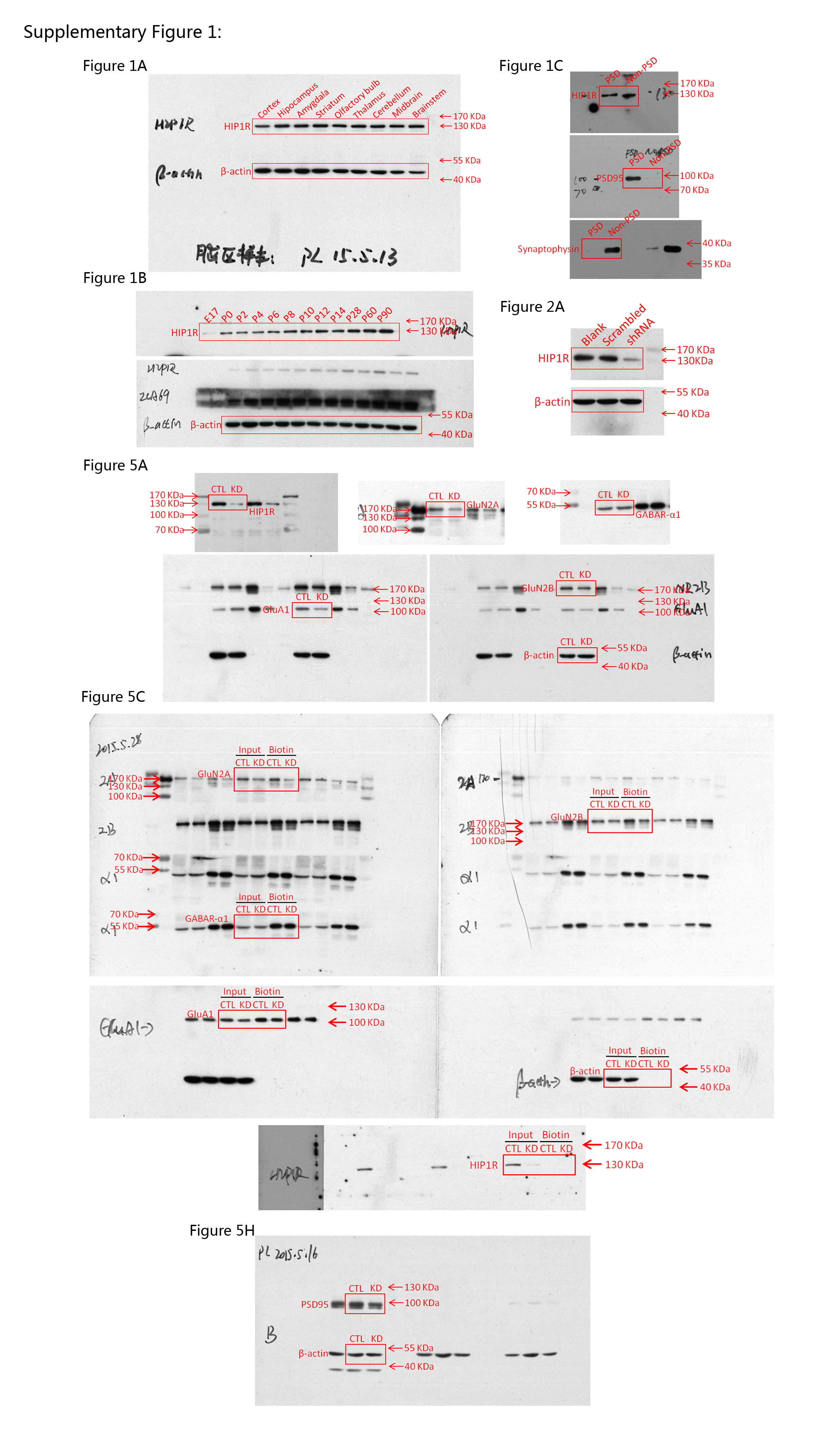

Supplement: FIGURE S1 — All raw blots show both molecular weights and the blot insets for each main figure. [file Image_1.tif]
